# Jian-Pi-Yi-Shen formula enhances perindopril inhibition of chronic kidney disease progression by activation of SIRT3, modulation of mitochondrial dynamics, and antioxidant effects

**DOI:** 10.1042/BSR20211598

**Published:** 2021-10-22

**Authors:** Xinhui Liu, Ruyu Deng, Xian Wei, Yuzhi Wang, Jiali Weng, Yunlan Lao, Jiandong Lu, Guoliang Xiong, Shunmin Li

**Affiliations:** 1 Department of Nephrology, Shenzhen Traditional Chinese Medicine Hospital, Guangzhou University of Chinese Medicine, Shenzhen, Guangdong, China; 2 Shenzhen Traditional Chinese Medicine Hospital Affiliated to Nanjing University of Chinese Medicine, Shenzhen, Guangdong, China; 3 The Fourth Clinical Medical College, Guangzhou University of Chinese Medicine, Shenzhen, Guangdong, China

**Keywords:** chronic kidney disease, Jian-Pi-Yi-Shen formula, mitochondrial dynamics, oxidative stress, perindopril erbumine, sirtuins

## Abstract

Chronic kidney disease (CKD) is a global public health problem. Renin–angiotensin system (RAS) blockade is the mainstay of CKD therapy with limitations. Jian-Pi-Yi-Shen formula (JPYSF) is a traditional herbal decoction and has been used for treating CKD for decades. The purpose of the present study was to investigate the intervention effects of combined used of perindopril erbumine (PE) and JPYSF on CKD progression and explore their underlying mechanisms. CKD rat model was induced by feeding a diet containing 0.75% *w/w* adenine for 3 weeks. CKD rats were treated with PE or JPYSF or PE+JPYSF from the induction of CKD and lasted 4 weeks. Renal function was evaluated by serum creatinine (Scr) and blood urea nitrogen (BUN). Pathological lesions were observed by Periodic acid–Schiff (PAS) and Masson’s trichrome staining. The protein expression was tested by Western blot and immunohistochemistry analysis. The morphology of mitochondria was observed by transmission electron microscope. The results showed that combined used of PE and JPYSF could better improve renal function and pathological lesions and ameliorate renal fibrosis in CKD rats. Administration of PE and JPYSF enhanced sirtuin 3 (SIRT3) expression, inhibited mitochondrial fission, promoted mitochondrial fusion, and suppressed oxidative stress in the kidney of CKD rats. In conclusion, combined use of PE and JPYSF protected against CKD more effectively than either alone. The underlying mechanism may be associated with activation of SIRT3, modulation of mitochondrial dynamics, and antioxidant effects.

## Introduction

Chronic kidney disease (CKD) is a common non-communicable disease that has become a significant public health concern. CKD has a high global prevalence with a consistent estimated global CKD prevalence of between 11 and 13% with the majority stage 3 [1]. CKD is an irreversible disease, and it is always associated with cardiovascular comorbidities and premature death. As CKD progresses to end-stage kidney disease (ESKD), patients need renal replacement therapy in the form of dialysis or kidney transplantation to maintain their lives. Globally, it is estimated that more than 3 million ESKD patients are receiving dialysis therapy [2,3]. Despite the global burden and costs of CKD are immense, current therapeutic approaches for CKD are still limited. Hence, there is an urgent need to explore the optimal treatment strategies to retard CKD progression.

Renin–angiotensin system (RAS) blockade is the mainstay of CKD therapy especially for diabetic kidney disease and hypertensive kidney disease, including angiotensin-converting enzyme inhibitor (ACEI) and angiotensin receptor blocker (ARB) [4,5]. Although the application of RAS inhibitors has delayed the progress of CKD in the past decades, the global incidence of ESKD has continued to increase [6]. Moreover, the renal protective effects of RAS inhibitors in different subgroups of CKD patients are not the same [7], and its application is also controversial with the decline of renal function [8]. People in many countries around the world use traditional medicinal plants to treat diseases [9]. The use of traditional Chinese herbal medicine (TCHM) has been a major complementary and alternative branch of CKD therapy due to its clinical efficacy and safety [10,11]. Jian-Pi-Yi-Shen formula (JPYSF) is a traditional herbal decoction and has been used for treating CKD for decades. Our previous studies have revealed that treatment with JPYSF could ameliorate CKD progression in CKD rat model [12,13]. However, whether the combination of JPYSF and RAS inhibitor can exert better renal protective effect remains unknown.

Sirtuin 3 (SIRT3) is a nicotinamide adenine dinucleotide (NAD^+^)-dependent protein deacetylase, mainly localizes in the mitochondrial matrix and plays an important role in regulating mitochondrial function, such as tricarboxylic acid cycle, fatty acid oxidation, oxidative phosphorylation, reactive oxygen species (ROS) detoxification, mitochondrial dynamics etc [14]. The SIRT3 protein is highly expressed in mitochondria-rich tissues including kidney [15]. In addition, mitochondrial dynamics and oxidative stress are involved in the pathogenesis of various kidney diseases [16,17]. The present study was designed to investigate the intervention effects of the combination of JPYSF and ACEI on CKD progression and explore their underlying mechanisms from the perspective of SIRT3, mitochondrial dynamics, and oxidative stress.

## Materials and methods

### Preparation of JPYSF extract

JPYSF is composed of eight traditional medicinal plants (Table 1). The plant names have been validated with https://mpns.science.kew.org/mpns-portal/. The raw herbs were boiled in ddH_2_O, filtered, concentrated, and freeze-dried as described previously [12]. The chemical constituents presented in JPYSF extract have been characterized by using ultra-performance liquid chromatography with quadrupole time-of-flight tandem mass spectrometry (UPLC-Q-TOF-MS/MS) in our previous study. A total of 71 compounds were tentatively identified from JPYSF extract, including saponins, flavonoids, sesquiterpenoids, coumarins, phenylpropanoids, anthranones, anthraquinones, tannins, phenolic acids, and others [18].

**Table 1:** The herbal composition of JPYSF

Plant name	Chinese name	Medicinal part	Dosage
Astragalus mongholicus Bunge	Huang Qi	Root	30 g
Atractylodes macrocephala Koidz.	Bai Zhu	Rhizome	10 g
Dioscorea oppositifolia L.	Shan Yao	Rhizome	30 g
Cistanche deserticola Ma	Rou Cong Rong	Fleshy stem	10 g
Wurfbainia compacta (Sol. ex Maton) Skornick. and A.D. Poulsen	Bai Dou Kou	Fruit	10 g
Salvia miltiorrhiza Bunge	Dan Shen	Root and rhizomes	15 g
Rheum palmatum L.	Da Huang	Root and rhizomes	10 g
Glycyrrhiza glabra L.	Zhi Gan Cao	Root and rhizomes	6 g

### Chemicals and antibodies

Adenine and perindopril erbumine (PE) were purchased from MedChemExpress (Shanghai, China). The primary antibodies included rabbit anti-fibronectin (FN), rabbit anti-type IV collagen (Col-IV) (Abcam, Cambridge, MA, U.S.A.), rabbit anti-SIRT3, mouse anti-optic atrophy 1 (OPA-1), rabbit anti-superoxide dismutase 1 (SOD1), rabbit anti-NADPH oxidase 2 (NOX2), rabbit anti-NOX4, mouse anti-glyceraldehyde-3-phosphate dehydrogenase (GAPDH) (Proteintech, Wuhan, China), rabbit anti-dynamin-related protein 1 (Drp-1), rabbit anti-SOD2, and rabbit anti-catalase (Cell Signaling Technology, Beverly, MA, U.S.A.). Horseradish peroxidase (HRP)-conjugated secondary antibodies were purchased from Life Technologies (Carlsbad, CA, U.S.A.).

### Animals and experimental design

Healthy male Spraque–Dawley (SD) rats weighted 150–180 g were obtained from Guangdong Medical Laboratory Animal Center (Foshan, China). After 1 week of acclimatization, all rats were randomly divided into the following five groups with six rats per group: control group, CKD group, CKD+PE group, CKD+JPYSF group, and CKD+PE+JPYSF group. CKD was induced by feeding a diet containing 0.75% *w/w* adenine for 3 weeks. PE and JPYSF were administered by gastric irrigation at the dose of 0.42 and 10.89 g/kg/day, respectively. The treatment started with induction of CKD and lasted 4 weeks. At the end of the study, all rats were anesthetized with sodium pentobarbital (50 mg/kg body weight, intraperitoneal injection), and blood samples were obtained from abdominal aorta. The rats were killed by cervical dislocation without regaining consciousness. Kidneys were rapidly harvested for further analysis. All animal experiments were carried out in Shenzhen Traditional Chinese Medicine Hospital, Guangzhou University of Chinese Medicine and were conducted with protocols approved by the Experimental Animal Ethics Committee of Guangzhou University of Chinese Medicine (No. 20190226020).

### Measurement of renal function indexes

Serum creatinine (Scr) and blood urea nitrogen (BUN) were used to evaluate renal function. They were measured by a creatinine serum detection kit and a BUN detection kit (StressMarq Biosciences, British Columbia, Canada), respectively, following the manufacturer’s instructions. In brief, standards, samples, and color reagents were pipetted into 96-well plates in sequence, and the optical density values at 490 nm for Scr and 450 nm for BUN were read, respectively.

### Histopathology

The kidney tissues were dehydrated, embedded in paraffin, and cut into 4-µm-sections. Periodic acid–Schiff (PAS) staining was used to detect renal tubular injury, and Masson’s trichrome staining was used to evaluate tubular interstitial fibrosis. All sections were dewaxed and rehydrated before staining. For PAS staining, after oxidization in 0.5% periodic acid solution for 5 min, the sections were treated with Schiff’s reagent for 15 min and counterstained with Hematoxylin. For Masson’s trichrome staining, after 5–10 min of Weiger’s iron Hematoxylin staining, the slices were treated with Ponceau acid–fuchsin solution, 1% phosphomolybdic acid, 2% Aniline Blue, and 1% glacial acetic acid in sequence.

### Western blotting

Proteins of kidney cortex were extracted by using RIPA lysis buffer and quantified using the Bradford method. Equivalent amounts of protein were separated on 7 or 10% SDS/PAGE gels and then transferred to nitrocellulose membranes (Millipore, Billerica, MA, U.S.A.). The membranes were blocked with 5% non-fat milk and then incubated with primary antibodies at 4°C overnight. After incubation with HRP-conjugated secondary antibodies and chemiluminescent HRP substrate, the blots were visualized and quantified using ChemiDoc MP Imaging System (Bio-Rad Laboratories, Hercules, CA, U.S.A.).

### Immunohistochemistry

The paraffin-embedded kidney tissues were cut into 6-µm-sections. After dewaxing and antigen retrieval, the slides were immersed in 3% hydrogen peroxide to blunt endogenous peroxidase activity, and then blocked with 10% goat serum for 1 h at 37°C. Subsequently, they were incubated with primary antibodies at 4°C overnight. Following treatment with SignalStain Boost Detection Reagent (Cell Signaling Technology), the slides were developed with SignalStain diaminobenzidine (DAB) substrate (Cell Signaling Technology) to produce a brown product. The integrated optical density (IOD) values of the positive staining areas were measured by ImagePro Plus 6.0 software (Media Cybernetics, CA, U.S.A.).

### Transmission electron microscopy

Kidney tissue blocks (∼1 mm^3^) were fixed in 2.5% glutaric dialdehyde at 4°C overnight and washed with 0.1 M PBS for three times, then post-fixed in 1% osmium tetraoxide for 2 h at room temperature. After being dehydrated in a graded series of ethanol concentrations, the tissue blocks were embedded in epoxy resin. Sections of 60–80 nm thickness were placed on 150 meshes cuprum grids that were double-stained with 2% uranyl acetate and 2.6% lead citrate. The cuprum grids were examined with a HT7700 transmission electron microscope (Hitachi, Tokyo, Japan).

### Statistical analysis

Data are expressed as the mean ± standard error of the mean (SEM). Statistically significant differences were determined by one-way ANOVA analysis and followed by Tukey’s *post hoc* test using GraphPad Prism 9 Software (San Diego, CA, U.S.A.). A value of *P*<0.05 was considered statistically significant difference.

## Results

### The effects of PE and JPYSF on renal function indexes in CKD

**Figure 1:**
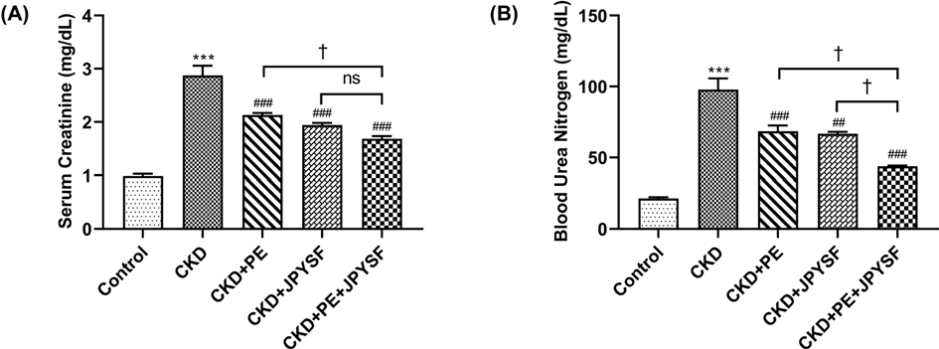
PE and JPYSF improved renal function in CKD rats (**A**) The levels of Scr in different groups. Compared with the control group, Scr level was markedly elevated in CKD rats and declined after PE or JPYSF treatment, especially in the combined treatment group. (**B**) The levels of BUN in different groups. PE, JPYSF and combined treatment reduced the BUN levels of CKD rats. Data are presented as the means ± SEM, *n*=4–6 rats per group (***P<0.001 compared with the control group; ^##^*P*<0.01, ^###^*P*<0.001 compared with the CKD group; ^†^*P*<0.05, compared with the CKD+PE+JPYSF group; ns, no significance).

Scr and BUN are two commonly used indicators of renal function. As shown in Figure 1A,B, the levels of Scr and BUN were both significantly increased in the CKD group compared with the control group (*P*<0.001). Either PE or JPYSF could significantly mitigate the increases in Scr and BUN in CKD rats (*P*<0.001). Of note, the combination of PE and JPYSF further reduced the levels of Scr and BUN compared with the CKD+PE or the CKD+JPYSF group (*P*<0.05, except for the comparison of Scr between the CKD+JPYSF and the CKD+PE+JPYSF group).

### The effects of PE and JPYSF on renal structure in CKD

**Figure 2:**
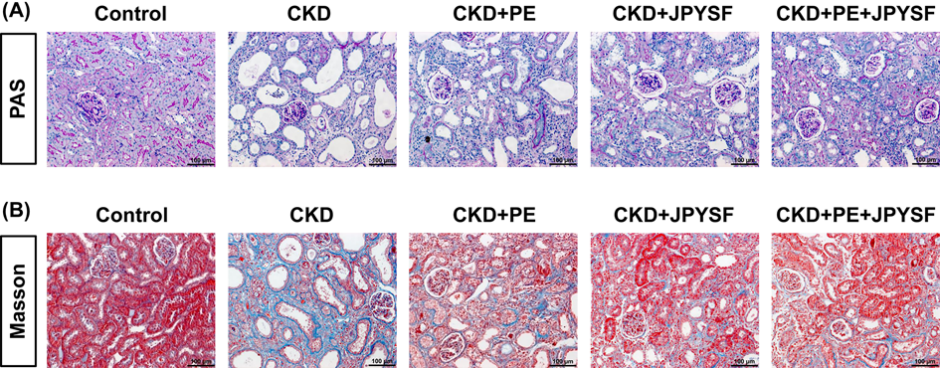
PE and JPYSF ameliorated renal pathological injury in CKD rats (**A**) Representative PAS-stained pictures of each group. (**B**) Representative Masson’s stained pictures of each group. Obvious renal tubular epithelial cells shedding and tubular dilation in PAS staining and large amounts of collagen fibrils deposition (blue staining) in Masson’s staining were observed in CKD rats. Treatment with PE or JPYSF, especially for combined therapy of PE and JPYSF, significantly ameliorated these pathological lesions. All images are shown at identical magnification, ×200, scale bar = 100 μm.

PAS staining showed obvious renal tubular epithelial cells shedding, tubular atrophy and dilation in CKD rats (Figure 2A). Masson’s staining displayed the deposition of large amounts of collagen fibrils (blue staining) in the renal interstitium of CKD rats (Figure 2B). Treatment with PE or JPYSF, especially for combined therapy of PE and JPYSF, significantly ameliorated these pathological lesions.

### The regulation of PE and JPYSF on fibrotic proteins expression in CKD

**Figure 3:**
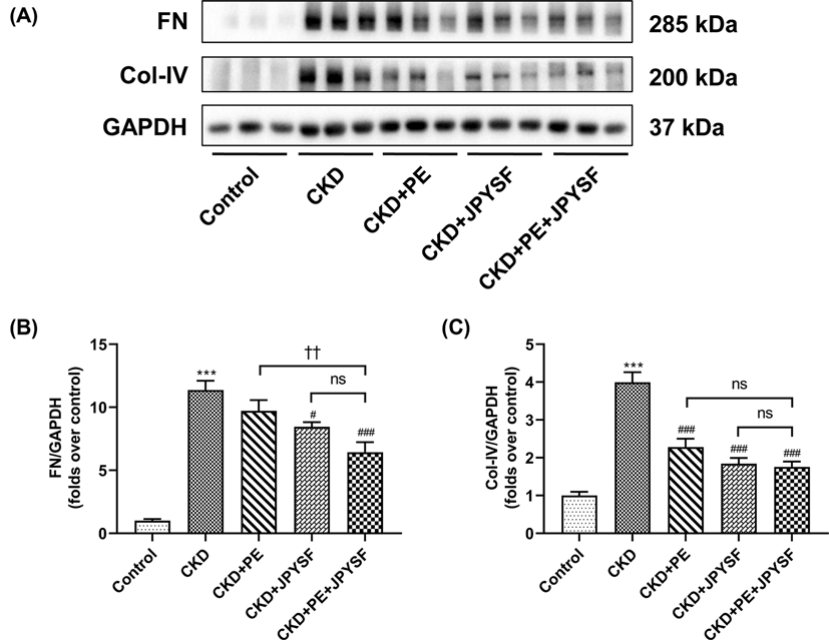
PE and JPYSF suppressed the expression of fibrotic markers in CKD rats (**A**) Representative Western blot images of FN and Col-IV. (**B**,**C**) Densitometric analysis of FN and Col-IV protein expression normalized to GAPDH content. The expression of FN and Col-IV were markedly up-regulated in the CKD group. Treatment with PE significantly reduced Col-IV but not FN expression. While treatment with JPYSF or combined use of PE and JPYSF significantly down-regulated both FN and Col-IV expression. Data are presented as the means ± SEM, *n*=4–6 rats per group (***P<0.001 compared with the control group; ^#^*P*<0.05, ^###^*P*<0.001 compared with the CKD group; ^††^*P*<0.01, compared with the CKD+PE+JPYSF group; ns, no significance).

Renal fibrosis is characterized by deposition of extracellular matrix, such as FN and Col-IV. As shown in Figure 3, the expression of FN and Col-IV were markedly up-regulated in the CKD group (*P*<0.001). Treatment with PE significantly reduced Col-IV but not FN expression. While treatment with JPYSF or combined use of PE and JPYSF significantly down-regulated both FN and Col-IV expression.

### The regulatory effects of PE and JPYSF on SIRT3 in CKD

**Figure 4:**
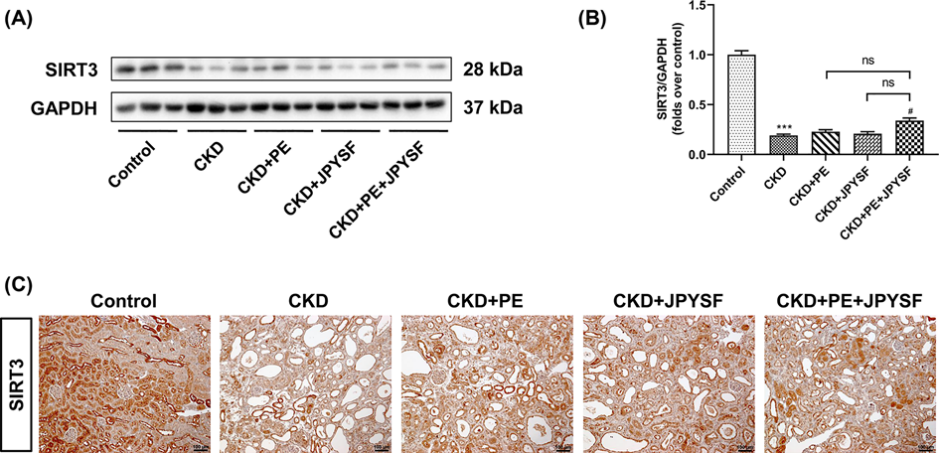
PE and JPYSF enhanced the expression of SIRT3 in CKD rats (**A**) Representative Western blot images of SIRT3. (**B**) Densitometric analysis of SIRT3 protein expression normalized to GAPDH content. (**C**) Representative IHC images of SIRT3 in different groups. The expression of SIRT3 was significantly reduced in the CKD group. The combination of PE and JPYSF could significantly up-regulate the expression of SIRT3. All images are shown at identical magnification, ×100, scale bar = 100 μm. Data are presented as the means ± SEM, *n*=4–6 rats per group (***P<0.001 compared with the control group; ^#^*P*<0.05 compared with the CKD group; ns, no significance).

Western blot analysis showed that the expression of SIRT3 was significantly reduced in the CKD group (*P*<0.001). Treatment with PE or JPYSF alone cannot increase the expression of SIRT3, and only the combination of PE and JPYSF could significantly up-regulate the expression of SIRT3 (*P*<0.05, Figure 4A,B). This result was further verified by IHC analysis (Figure 4C).

### The effects of PE and JPYSF on mitochondrial dynamics in CKD

**Figure 5:**
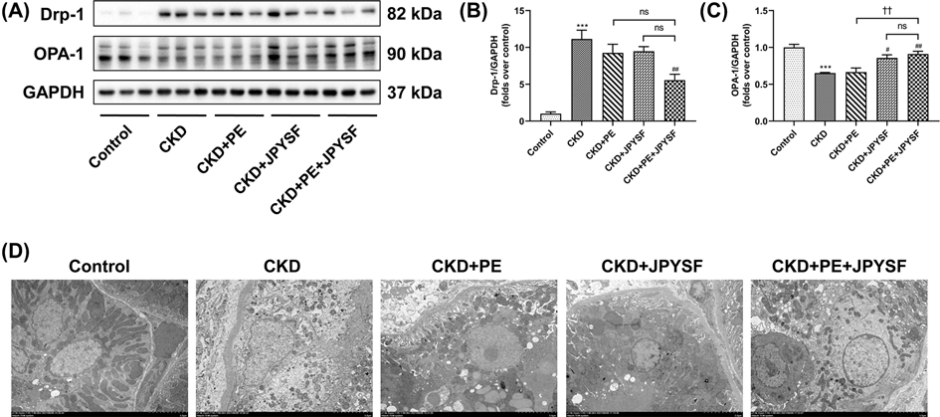
PE and JPYSF modulated mitochondrial dynamics in CKD rats (**A**) Representative Western blot images of Drp-1 and OPA-1. (**B**,**C**) Densitometric analysis of Drp-1 and OPA-1 protein expression normalized to GAPDH content. Drp-1 was markedly up-regulated in CKD rats. In contrast, the expression of OPA-1 was significantly down-regulated in CKD rats. Only combined PE and JPYSF treatment could significantly reverse the expression trend of both Drp-1 and OPA-1. (**D**) Representative TEM images of mitochondria in the kidneys from different groups. Mitochondria fragmented into small, punctuated suborganelles in CKD rats, and this effect was partially reversed in the CKD+PE+JPYSF group. All images are shown at identical magnification, scale bar = 5 μm. Data are presented as the means ± SEM, *n*=4–6 rats per group (***P<0.001 compared with the control group; ^#^*P*<0.05, ^##^*P*<0.01 compared with the CKD group; ^††^*P*<0.01, compared with the CKD+PE+JPYSF group; ns, no significance).

Mitochondrial dynamics is governed by fission and fusion. Drp-1, a master regulator of mitochondrial fission, was markedly up-regulated in CKD rats. In contrast, the expression of OPA-1, a mediator of mitochondrial fusion, was significantly down-regulated in CKD rats (*P*<0.001). Only combined PE and JPYSF treatment could significantly reverse the expression trend of both Drp-1 and OPA-1 (Figure 5A–C). Moreover, transmission electron microscopy (TEM) showed that mitochondria fragmented into small, punctuated suborganelles in CKD rats, and this effect was partially reversed in the CKD+PE+JPYSF group (Figure 5D).

### The effects of PE and JPYSF on oxidative stress in CKD

**Figure 6:**
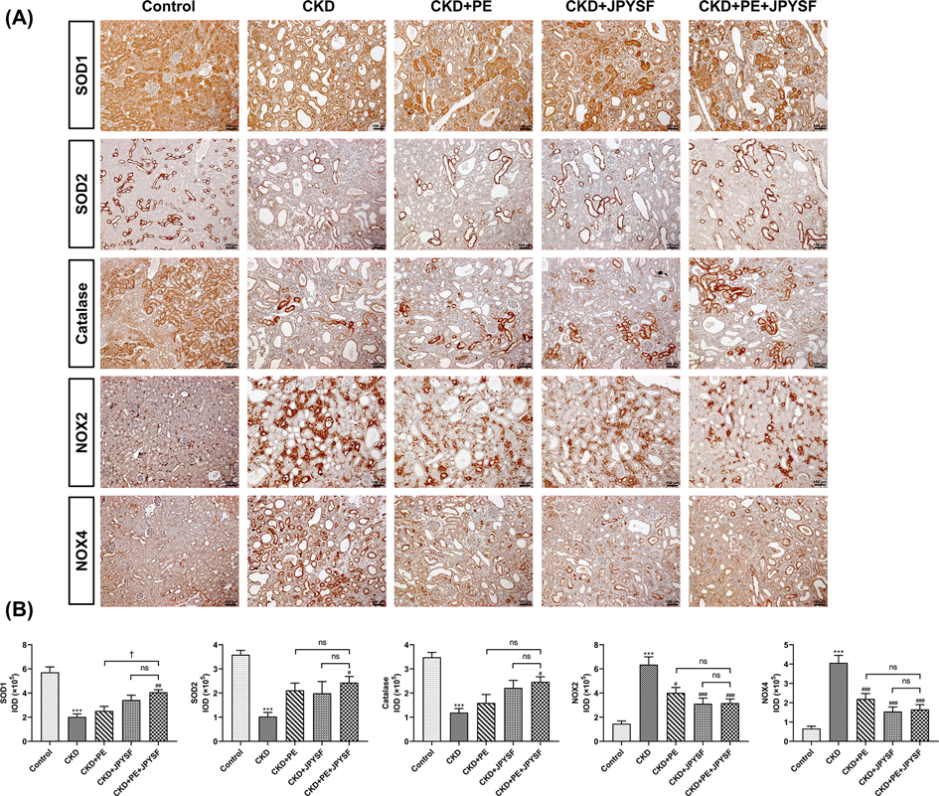
PE and JPYSF suppressed oxidative stress in CKD rats (**A**) Representative IHC images of SOD1, SOD2, catalase, NOX2 and NOX4 in different groups. (**B**) Quantitative analysis of positive staining areas for SOD1, SOD2, catalase, NOX2 and NOX4. Only combination of PE and JPYSF could significantly up-regulate the expression of SOD1, SOD2, and catalase. Treatment with PE or JPYSF or combined treatment could inhibit NOX2 and NOX4 expression in CKD rats. All images are shown at identical magnification, ×100, scale bar = 100 μm. Data are presented as the means ± SEM, *n*=5 rats per group (***P<0.001 compared with the control group; ^#^*P*<0.05, ^##^*P*<0.01, ^###^*P*<0.001 compared with the CKD group; ^†^*P*<0.05, compared with the CKD+PE+JPYSF group; ns, no significance).

Oxidative stress is caused by the imbalance between the production of free radicals and the antioxidant system. As shown in Figure 6A, the expression of SOD1, SOD2 and catalase, three antioxidant enzymes, were sharply reduced in the kidney of CKD rats. However, the expression of NOX2 and NOX4, two critical enzymes involved in ROS formation, were obviously increased in the kidney of CKD rats. Quantitative analyses revealed that only combination of PE and JPYSF could significantly up-regulate the expression of SOD1, SOD2 and catalase. PE or JPYSF or combined treatment could inhibit NOX2 and NOX4 expression in CKD rats (*P*<0.05, Figure 6B).

## Discussion

In the present study, combined used of PE and JPYSF improved renal function and pathological lesions and ameliorated renal fibrosis in CKD rats. In mechanism, administration of PE and JPYSF enhanced SIRT3 expression, modulated mitochondrial dynamics, and inhibited oxidative stress in the kidney of CKD rats.

Currently, RAS inhibitors are the mainstay of treatment for CKD. Clinical trials have shown that prescription of ACEI or ARB could reduce albuminuria and slow the progression of diabetic nephropathy [19]. In clinical practice guidelines, RAS inhibitors are recommended as the first-line antihypertensive drug of choice for patients with CKD with or without diabetes [20]. Moreover, RAS inhibitors have also showed cardiovascular protection beyond blood pressure control. However, the beneficial effects of RAS inhibitors for patients with advanced CKD remain controversial [8,21]. The evidences in favor of RAS inhibitors are less strong for patients with lower levels of albuminuria (30–300 mg/g creatinine), especially for CKD patients without diabetes [22,23]. In addition, the safety of long-term use of RAS inhibitors and acute reduction in glomerular filtration rate (GFR) following RAS inhibitors are worrying [7]. Therefore, many patients seek out alternative therapies such as TCHM to treat CKD [24]. In fact, TCHM is frequently used in conjunction with RAS inhibitors for treatment of CKD in China and many other Asian countries [25]. In the present study, combination of PE and JPYSF showed better efficacy in delaying CKD progression as evidenced by preserving renal function and structure and ameliorating renal fibrosis (Figures 1-3). Additional data showed that combination of PE and JPYSF significantly down-regulated angiotensin-converting enzyme (ACE) expression in CKD rats (Supplementary Figure S1), which suggested that JPYSF can act on RAS.

SIRT3 is mainly localized in the mitochondrial matrix. Accumulating evidence suggests that SIRT3 is a master regulator of mitochondrial function by activating multiple targets involved in ATP production, energy metabolism, antioxidant activity, and mitochondrial dynamics [26]. Mitochondria are dynamic organelles, continuously undergoing the process of fission and fusion [27]. Fission depends on cytosolic Drp-1 and its receptors on the outer mitochondrial membrane. Fusion is mediated by OPA-1 and mitofusins [28]. Recent studies revealed that SIRT3 was a new regulator of mitochondrial dynamics [29]. Mice with cisplatin-induced AKI had remarkable SIRT3 reduction that induced the recruitment of Drp-1 on mitochondrial membranes and down-regulation of OPA-1, ultimately tipping mitochondrial dynamics toward fission and fragmentation [30]. Similar with this study, our results also showed reduced SIRT3 and increased mitochondrial fission in the kidney of adenine-induced CKD rat model (Figures 4 and 5). SIRT3 exerts antioxidative stress effect through two mechanisms. It prevents excessive electron leakage and ROS production by regulating the function of complexes I and III. Moreover, it contributes to superoxide discharge through deacetylation and activation of SOD2 [31]. In addition, SIRT3 can also induce the transcription of SOD2 and catalase by deacetylating forkhead box O3A [32]. In the kidney, mitochondria and the NOX family are the major sources of endogenous ROS [33]. During the course of CKD, there are a series of dynamic events that may initially increase mitochondrial ROS production but not able to maintain it as the disease progresses [34]. Moreover, there is interplay between these two ROS sources that can lead to feed–forward mechanisms where activation of one source of ROS can lead to activation of another [35]. Previous study has shown that elevated NOX4 expression increased mitochondrial ROS production [36]. In this study, we only tested the expression of NOX and antioxidant enzymes. The source and clearance mechanism of ROS in this animal model need further research.

The limitations of the present study were that we did not detect ROS content in fresh kidney samples as direct evidence of oxidative stress and we did not measure the blood pressure of rats.

In conclusion, our study demonstrated that combined use of JPYSF and PE protected against CKD more effectively than either alone. The underlying mechanism may be associated with activation of SIRT3, modulation of mitochondrial dynamics, and antioxidant effects.

## Supplementary Material

Supplementary Figures S1-S5

## Data Availability

The data presented in the present study are available on request from the corresponding authors.

## Abbreviations

ACEI: angiotensin-converting enzyme inhibitor
ARB: angiotensin receptor blocker
BUN: blood urea nitrogen
CKD: chronic kidney disease
Col-IV: type IV collagen
Drp-1: dynamin-related protein 1
ESKD: end-stage kidney disease
FN: fibronectin
HRP: horseradish peroxidase
JPYSF: Jian-Pi-Yi-Shen formula
NAD: nicotinamide adenine dinucleotide
NOX: NADPH oxidase
OPA-1: optic atrophy 1
PAS: Periodic acid–Schiff
PE: perindopril erbumine
RAS: renin–angiotensin system
ROS: reactive oxygen species
Scr: serum creatinine
SIRT3: sirtuin 3
SOD: superoxide dismutase
TCHM: traditional Chinese herbal medicine
TEM: transmission electron microscopy
