# Supplementary material for: Jian-Pi-Yi-Shen formula enhances perindopril inhibition of chronic kidney disease progression by activation of SIRT3, modulation of mitochondrial dynamics, and antioxidant effects
Source: Biosci Rep. 2021 Oct 22;41(10):BSR20211598. doi: 10.1042/BSR20211598 (PMC8536834; doi:10.1042/BSR20211598)
Supplement: Supplementary Figures S1-S5 [file BSR-2021-1598_supp.pdf]

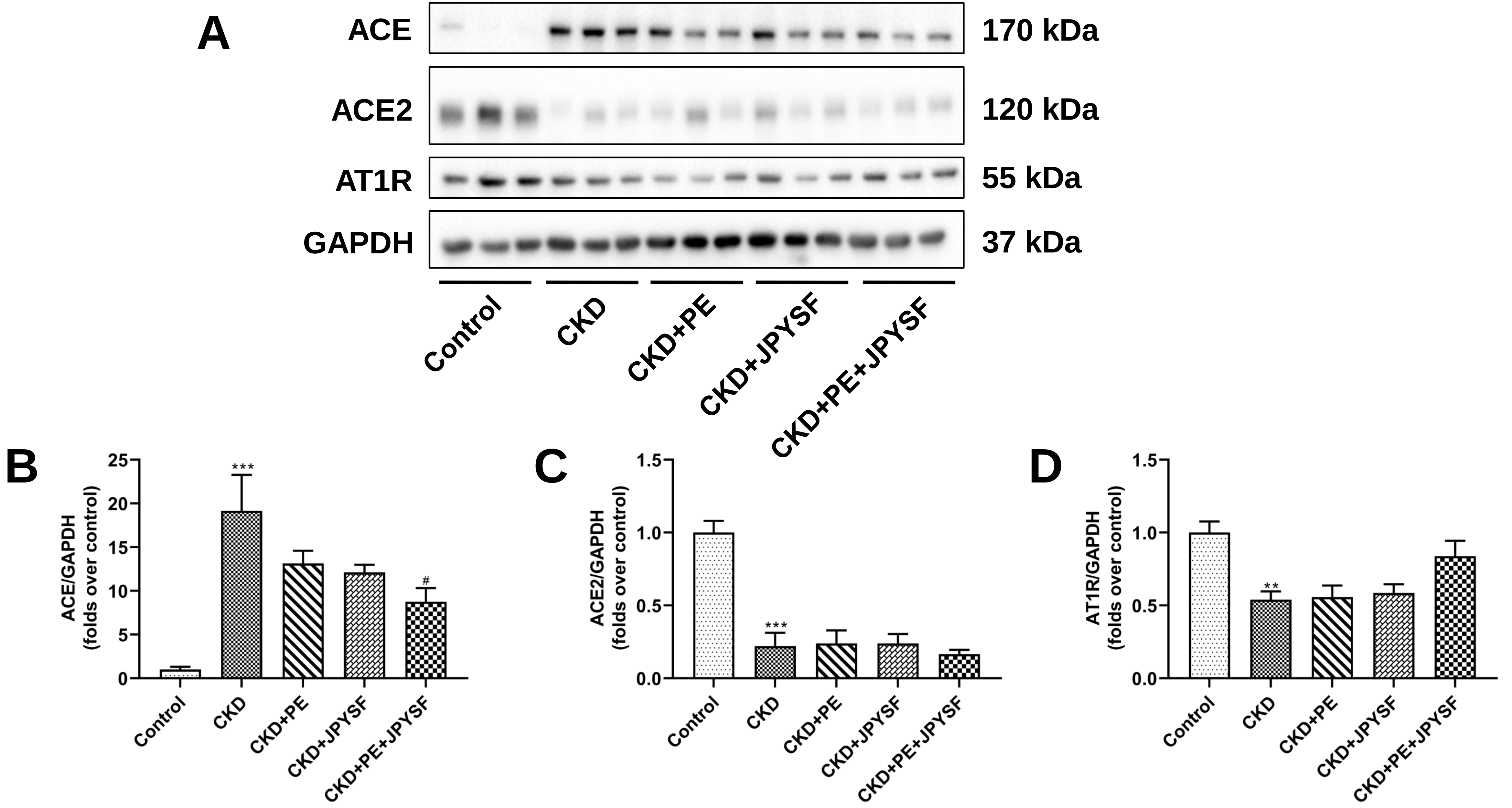

Supplementary Figure 1. The expression of RAS components in the kidney of each group. (A) Representative Western blot images of ACE, ACE2 and AT1R. (B-D) Densitometric analysis of ACE, ACE2 and AT1R protein expression normalized to GAPDH content. Data are presented as the means ± SEM (n=6, $^{***}P<0.001$, $^{**}P<0.01$ compared with the control group; $^{\#}P<0.05$ compared with the CKD group).

Supplementary Figure 2. Original western blot bands shown in Fig. 3A

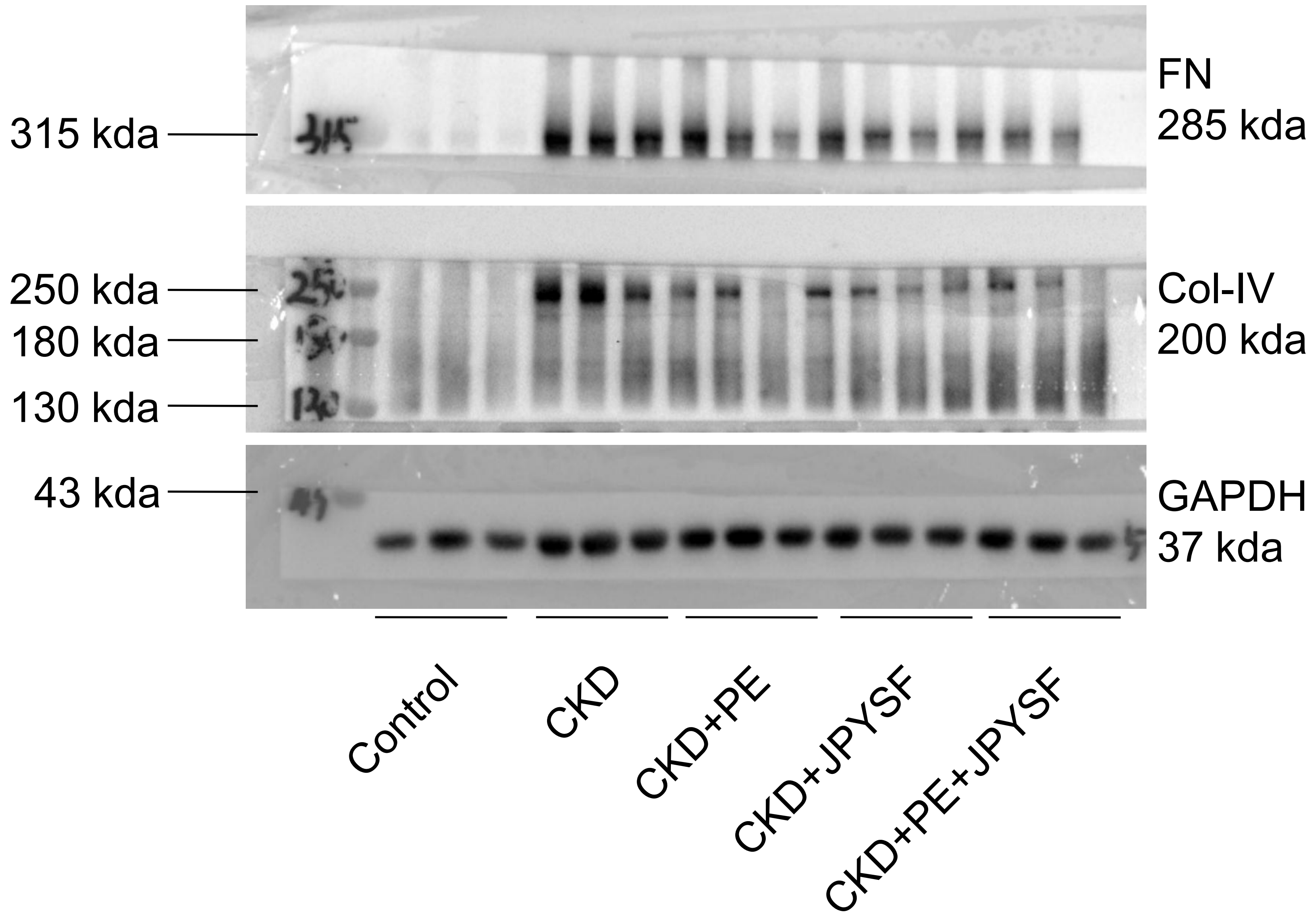

Marker:

Spectra™ Multicolor High Range Protein Ladder，#26625, Thermo Scientific

Primary antibodies:

1. FN, Rabbit polyclonal，abcam, 1:500
2. Col-IV, Rabbit polyclonal, abcam, 1:500
3. GAPDH, Mouse monoclonal, Proteintech, 1:5000

Supplementary Figure 3. Original western blot bands shown in Fig. 4A

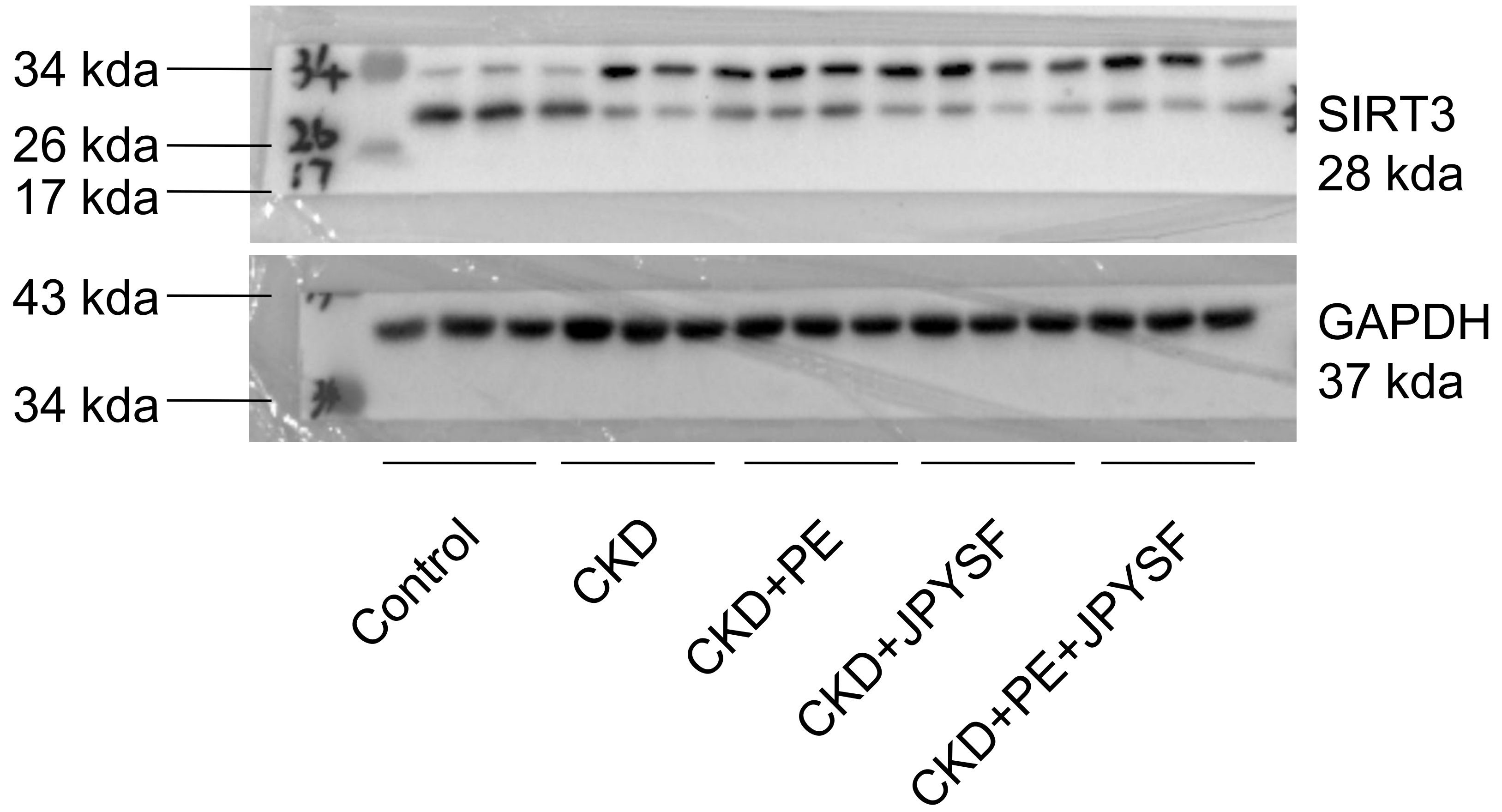

Marker:

PageRuler™ Prestained Protein Ladder, 10 to 180 kDa, 26616, Thermo Scientific

Primary antibodies:

1. SIRT3, Rabbit polyclonal , Proteintech, 1:500
2. GAPDH, Mouse monoclonal, Proteintech, 1:5000

Supplementary Figure 4. Original western blot bands shown in Fig. 5A

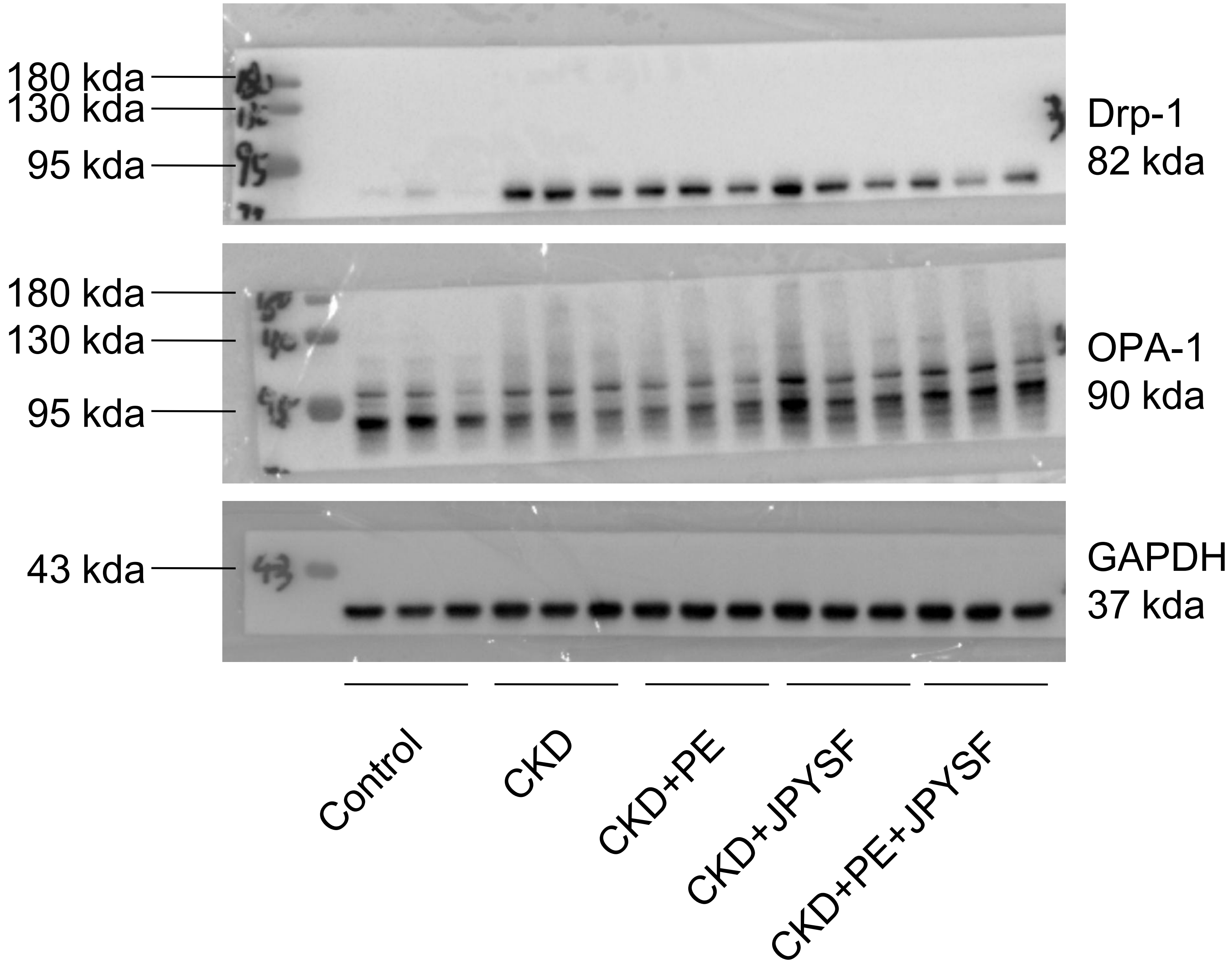

Marker:

PageRuler™ Prestained Protein Ladder, 10 to 180 kDa, 26616, Thermo Scientific

Primary antibodies:

1. Drp-1, Rabbit monoclonal , CST, 1:1000
2. OPA-1, Rabbit polyclonal, Proteintech, 1:1000
3. GAPDH, Mouse monoclonal, Proteintech, 1:5000

Supplementary Figure 5. Original western blot bands shown in Fig. S1A

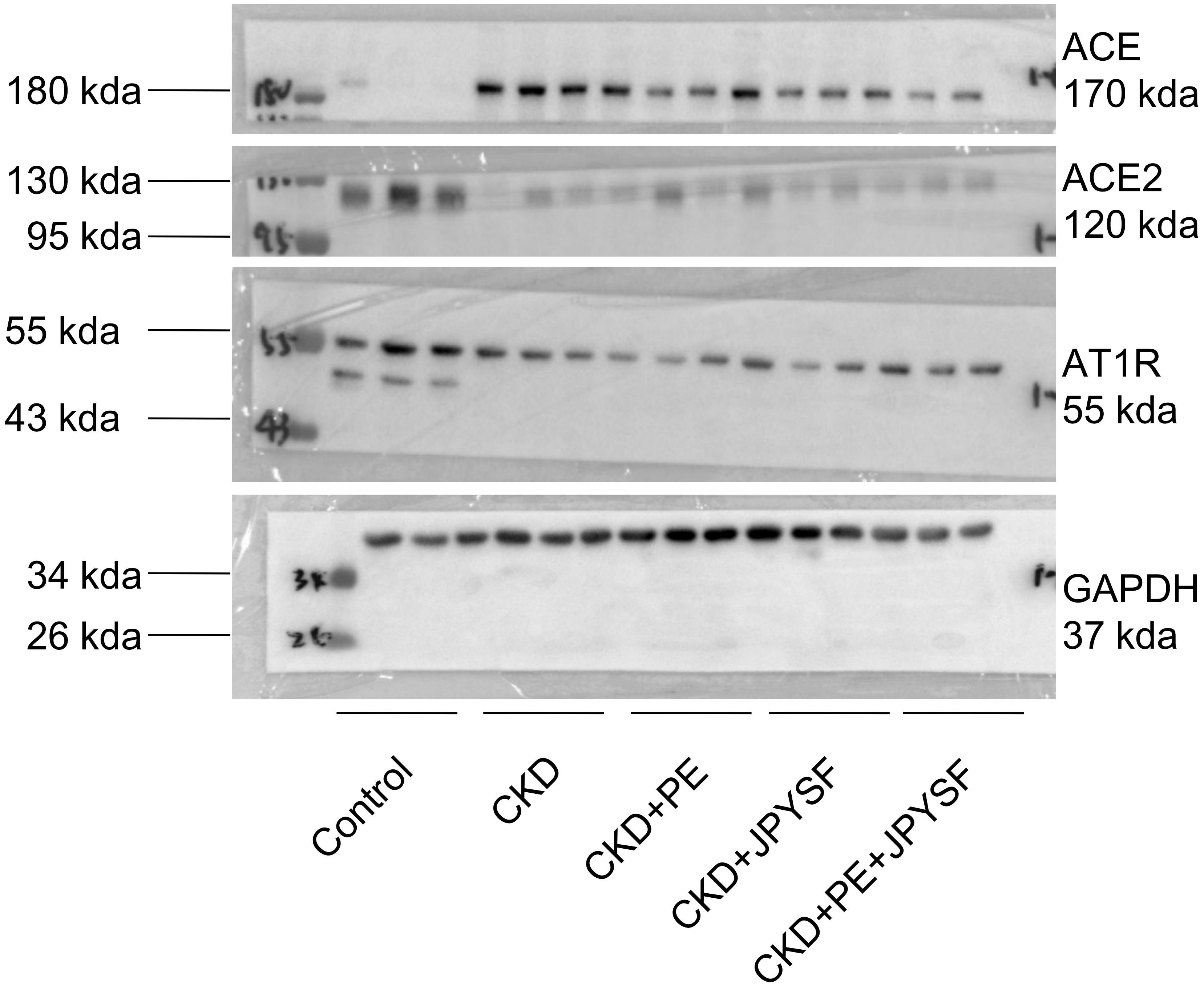

Marker:

PageRuler™ Prestained Protein Ladder, 10 to 180 kDa, 26616, Thermo Scientific

Primary antibodies:

1. ACE, Rabbit polyclonal, Proteintech, 1:500
2. ACE2, Rabbit polyclonal, Proteintech, 1:500
3. AT1R, Rabbit polyclonal, Proteintech, 1:500
4. GAPDH, Mouse monoclonal, Proteintech, 1:5000
